# Evidence of mental health-related morbidities and its association with socio-economic status among previously hospitalized patients with symptoms of COVID-19 in Bangladesh

**DOI:** 10.3389/fpubh.2023.1132136

**Published:** 2023-02-24

**Authors:** Asibul Islam Anik, Tanvir Ahmed, Ahmed Jojan Nandonik, Anwar Parvez, Shangjucta Das Pooja, Zarina Nahar Kabir

**Affiliations:** ^1^Department of Research, Monitoring and Evaluation, SAJIDA Foundation, Dhaka, Bangladesh; ^2^Division of Nursing, Department of Neurobiology, Care Sciences and Society, Karolinska Institute, Stockholm, Sweden

**Keywords:** community psychiatry, Coronavirus, DASS-21, low- and middle-income countries, post-COVID, mental health, socio-economic status, social psychiatry

## Abstract

**Introduction:**

The long-term impact of COVID-19 on mental health, particularly in relation to socio-economic vulnerabilities, has received little attention. This study reports the prevalence of mental health-related symptoms among previously hospitalized patients after recovery from COVID-19, and its association with socio-economic status (SES).

**Methods:**

Data collection of this cross-sectional study was conducted during February–April 2021, among previously hospitalized patients with COVID-19 like symptoms, on average six months after their discharge from the hospital. Using DASS-21, a validated scale to document symptoms of depression, anxiety, and stress, information on mental health-related symptoms were recorded from 481 respondents along with sociodemographic and economic information through telephone interviews. Chi-square tests were performed to identify significant group differences. Multinomial logistic regression analyzed the association between the changes in socioeconomic characteristics and mental health-related symptoms. Relative index of inequality (RII), slope index of inequality (SII), and concentration index (CIX) were applied to capture relevant inequalities in relation to mental health-related symptoms.

**Results:**

Eleven percent of the respondents reported changes in employment status, nearly half changes in income and expenditure. Forty-five percent reported symptoms of depression, anxiety and/or stress, and 12% reported coexistence of all three symptoms. Women [Adjusted Odds Ratio, AOR: 2.95; 95% Confidence Interval, CI: 1.39–5.68], and those who reported changes in occupation [AOR: 3.04; 95% CI: 1.01–9.08] and expenditure [AOR: 2.46; 95% CI: 1.12–5.37] were more likely to report all three mental health-related symptoms compared to men and those without changes in occupation and expenditure. The older age group was less likely [AOR: 0.96; 95%CI: 0.93–0.99] to report coexistence of all three symptoms compared to their younger counterparts. Negative values of concentration index (CIX) indicate that any one mental health-related symptom was significantly concentrated among those with lower expenditure and poor SES.

**Conclusion:**

This study will help in addressing mental health-related challenges after recovery from COVID-19 among the identified vulnerable groups through relevant community-based and clinical response, including counseling services, in Bangladesh and similar LMIC contexts.

## 1. Background

The challenges of the COVID-19 pandemic are multidimensional (1). There is a considerable pool of evidence reporting on the acute phase of COVID-19 which primarily include physical suffering (2), socioeconomic suffering (3), and gap(s) in response by the health system (4). However, evidence on the long-term impact of COVID-19 or what can be termed as post-COVID-19 consequences is rather slim compared to its acute phase and is still emerging. Existing evidence indicates that post-COVID consequences, also known as long-COVID, encompass a range of signs and symptoms which notably affect human wellbeing (2, 5). Among those, mental health-related signs and symptoms account for a considerable share, both in high-income countries (HICs) and low- and middle-income countries (LMICs) (6, 7).

In the first year of the pandemic, there was a 28% increase in the prevalence of major depressive disorder and a 25% increase in anxiety disorders worldwide (8, 9). Studies on patients with COVID-19 3–6 months after hospitalization reported a range of mental health-related issues including depression (10), post-traumatic stress disorder (PTSD) symptoms (11), and mood disorder (12). A systematic review of patients with COVID-19 after recovery revealed that after being discharged from the hospital, newly developed insomnia was observed among 24–40% (13), depression among 9–66% (14, 15), anxiety among 30–39% (16) and PTSD among 10–15.4% (14, 15).

The long-term effects of the COVID-19 pandemic have been found to be associated with profound mental health-related challenges in addition and related to consequences for physical health, disrupted socio-cultural sphere and other macro-level dimensions (e.g., economic, political, etc.), both for the individual and community (5, 17). A recent review (18) identified some of the challenges faced in LMICs including lack of adequate mental health service providers, poor mental health literacy among the population, scarcity and disparity of resources and services for mental health, and poverty. These pre-existing factors coupled with added challenges during COVID-19 pandemic exacerbated the mental health status of population in LMICs. These additional challenges include fear of being infected with Coronavirus, increasing economic burden, and lack of mental healthcare provisions due to lockdown (18). In addition to that, empirical evidence mentioned other socio-economic factors including restricted physical mobility, lockdowns, school, and business shutdowns, losing jobs, disruptions of livelihood, as well as the social and economic fallout- all potentially stimulate sadness, anxiety, worry, depression (19), fear (20), anger, frustration, loneliness and stress (9, 19, 21) during the pandemic.

Being hospitalized with a serious disease was found to have a negative impact on the psychological wellbeing of a patient (22). Studies from Turkey (23), Pakistan (24), and Netherlands (22) reported that the mental health-related symptoms were higher for women, young, and those who were unmarried (24), had low education, became unemployed (22, 23), resided in urban regions (24), and were hospitalized for a long period (more than 16 days) (22). On the other hand, recent studies have shown that demographic and cognitive factors, such as- a high level of self-efficacy (25) and mental health literacy (25, 26), along with high education level, stable occupational status (25), and older age (27) were significantly associated with adopting protective behaviors against COVID-19 (25) and health promoting behaviors (27), as well as improving mental health status (26).

The LMICs, including Bangladesh, are expected to experience the challenges of long-term mental health-related morbidities in relation to the COVID-19 pandemic for decades to come (5, 8, 17, 28). Recent evidence reported that mental health-related morbidities among adults in Bangladesh ranged from 6.5 to 31.0% before the pandemic (29). During the COVID-19 pandemic, Bangladesh reported the highest prevalence of anxiety (52%) and depression (48%) among the South Asian countries followed by Pakistan (anxiety 50%, depression 41%) (30). Several studies have reported mental health-related symptoms during the pandemic among the general population (31, 32), and in specific cohorts including medical students (33), healthcare workers (34, 35), and school students (36). One study specifically reported mental health-related symptoms of patients with COVID-19 during their hospital stay (37). Additionally, the association between stress and economic losses due to COVID-19 among the general population of Bangladesh was also found in some studies (38, 39). However, there is a lack of information in Bangladesh regarding the long-term consequence of COVID-19 on mental health after recovery and how it may be related to changes in socio-economic characteristics and inequality.

Hence, the current study aimed to investigate the prevalence of mental health-related symptoms (i.e., depression, anxiety, and stress) among previously hospitalized patients with symptoms of COVID-19 and its association with changes in socioeconomic status and related inequality in Bangladesh.

## 2. Materials and methods

### 2.1. Study design and participants

**Figure 1:**
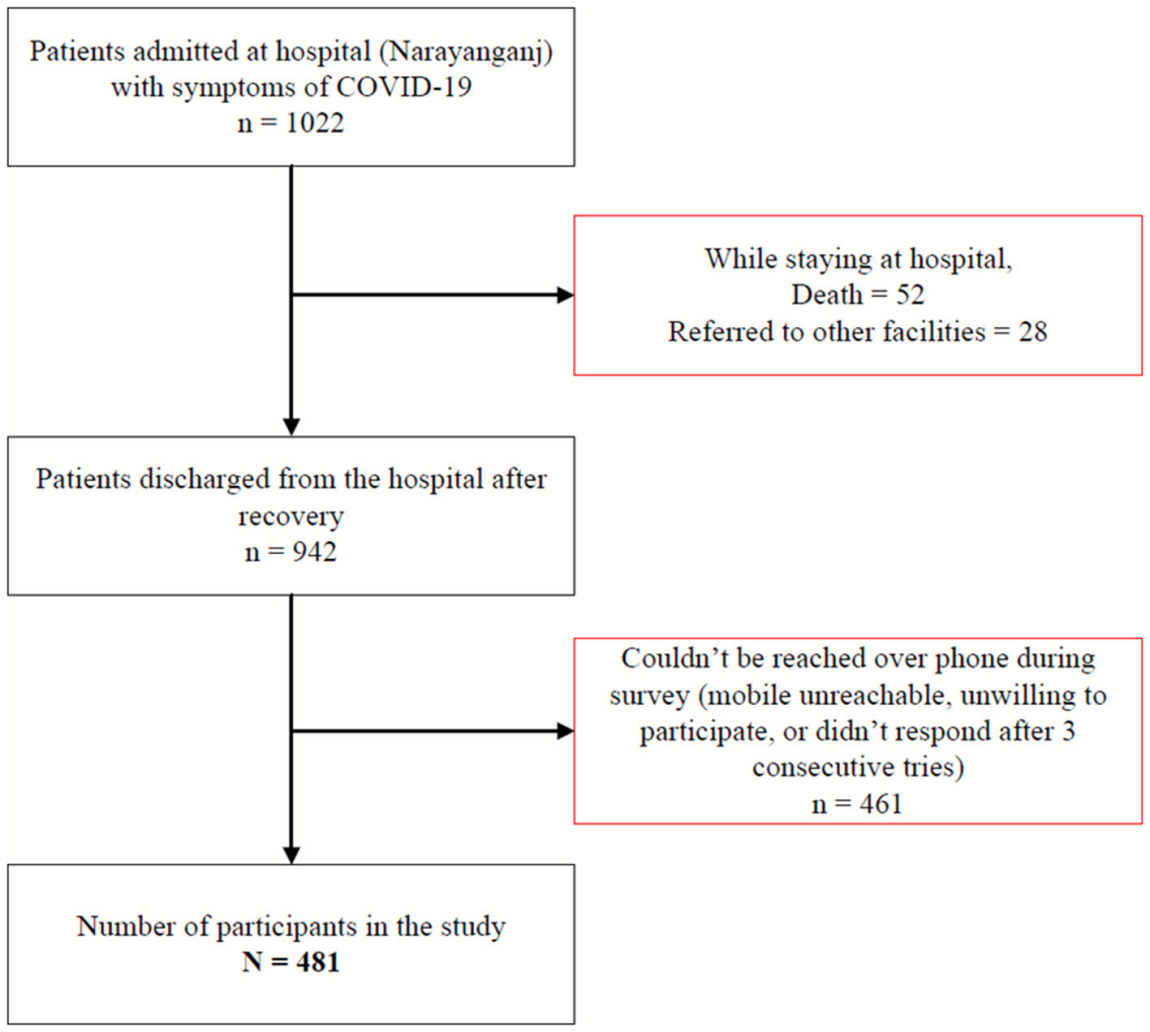
Sampling and sample size.

This cross-sectional survey included participants who had previously been hospitalized with COVID-19-like symptoms at a hospital dedicated only to patients with COVID-19. This hospital, run by a non-government humanitarian organization, was located in a peri-urban area of Narayanganj district (about 18 kilometers from the capital city Dhaka). Over a period of nine months (April–December 2020), 1,022 patients were admitted to the hospital with symptoms similar to COVID-19. The inclusion criteria of the study participants were: (i) patients who were admitted and received treatment at the COVID-19 dedicated hospital in Narayanganj; (ii) patients with symptoms of COVID-19 discharged from the hospital after recovery; and (iii) those aged 18 years or older. Participants were excluded from the study if they were unable to communicate due to mental or physical challenges. Of the 1022 patients admitted to the hospital, 52 died and 28 were referred to other facilities resulting in 942 who were discharged from the hospital after recovery. The non-response rate of the study was 49% including those who could not be reached over phone during the survey (*n* = 305) or did not want to participate (*n* = 156) in the study. Thus, the final sample size of the study was 481 (Figure 1).

### 2.2. Data collection procedure

The data collection was conducted in February–April 2021 amidst the ongoing waves of COVID-19 in the country. To adhere to restrictions of the time in terms of mobility and physical distancing, interviews were conducted over the telephone. An electronic survey questionnaire was prepared using a web-based survey application called “Kobo Toolbox” (40) and KoboCollect app for the data collection on tablets with the Android operating system. A team of seven data collectors and one supervisor with previous experience in conducting telephone surveys and using KoboCollect app were trained and also pretested the questionnaire. Each interview lasted about 30–40 min. To keep the non-response rate to the minimum level, each participant was reached out at least three times at different times on different days before considering the person as non-response. The data collection was conducted about 6 months after the discharge of the last patient from the hospital. The average gap between discharge and interview dates was 186 days.

### 2.3. Measurements

#### 2.3.1. Dependent variable

##### 2.3.1.1. Mental health-related symptoms

Mental health-related symptoms, expressed as symptoms of depression, anxiety, and stress, were assessed by the Depression, Anxiety, and Stress Scale (DASS) consisting of three sections (depression—DASS-D, anxiety—DASS-A and stress—DASS-S), popularly known as DASS-21 (41). Each section has seven statements. The participants were asked to rate their agreement to each statement on a four-point Likert scale ranging from 0 (did not apply to me at all) to 3 (applied to me very much). The responses of each statement in each section were summed and then multiplied by two. Thus the final score of each section is ranged from 0 to 42, and indicates the reported severity level based on depression, anxiety, and stress scores. DASS-21 scale is validated in Bangla by Alim et al. (42). In the current study, the Bangla version of DASS-21 was used and it showed high level of internal consistency in all three sections (Cronbach's alpha: depression = 0.80, anxiety = 0.72 and stress = 0.76). The overall reliability (alpha) was 0.90 indicating high internal consistency.

The severity index based on the total scores was: DASS-D 0–9 = no depression/normal, 10–13 = mild depression, 14–20 = moderate depression, 21–27 = severe depression and >27 = very severe depression; DASS-A 0–7 = no anxiety/normal, 8–9 = mild anxiety, 10–14 = moderate anxiety, 15–19 = severe anxiety and >19 = very severe anxiety; and DASS-S 0–14 = no stress/normal, 15–18 = mild stress, 19–25 = moderate stress, 26–33 = severe stress and >33 = very severe stress (41, 42). In this study, the severity indices of all three sections were converted into dichotomous variables; i.e., depressive symptoms no/yes (“no = 0”, if DASS-D≤9 vs. “yes = 1”, if DASS-D > 9), anxiety symptoms no/yes (“no = 0”, if DASS-A ≤ 7 vs. “yes = 1”, if DASS-A > 7), and stress symptoms no/yes (“no = 0”, if DASS-S ≤ 14 vs. “yes = 1”, if DASS > 14). Finally, all possible combinations of the dichotomous subscales were considered to obtain the main dependent variable “Symptoms of depression, anxiety and stress” as- “No symptoms at all”, “Any one symptom”, “Any two symptoms”, and “All three symptoms” (see Supplementary Figure 1).

#### 2.3.2. Independent variables

Background information included age in years (≤29, 30–39, 40–49, 50–59, and 60+), gender (male and female), household size (1–4, 5, 6, and 7+ members), type of residence (own house, rented house and other), completed years of education (0–5, 6–10, 11–12 and more than 12), occupation (currently in paid employment, not in paid employment and homemaker), monthly expenditure in Bangladeshi Taka (BDT) (in quintiles, i.e., ≤15,000, 15,001–25,000, 25,001–30,000, 30,001–50,000 and more than 50,000), the monthly income in BDT (in quintiles, i.e., ≤20,000, 20,001–30,000, 30,001–40,000, 40,001–60,000 and more than 60,000), and household socio-economic status (SES) (poor, middle and rich).

Household SES was created using a combination of three variables, i.e., monthly income, monthly expenditure, and household wealth-index. Information on the wealth index was based on questions (bivariate: yes/no) on household's ownership of a number of items (such as television, radio, mobile phone, telephone (landline), fridge, closet, fan, Instant Power Supply (IPS), Air Condition (AC), laptop, Digital Versatile Disc (DVD), pump, Compressed Natural Gas vehicles (CNG), rickshaw, bike, cycle, boat, motor boat, and car) and other characteristics that are related to wealth status (such as ownership of land, buffalo and farm). Each of these household assets for which information was collected was assigned a weight or factor score generated through principal components analysis (PCA). The resulting asset scores were further standardized in relation to a standard normal distribution with a mean of zero and a standard deviation of one. Then these standardized scores were divided into five equal quintiles with the first representing the poorest 20% and the fifth representing the richest 20%. After creating the variable “wealth-index”, other two variables “income” and “expenditure” were combined with “wealth-index” based on their mean values, and scores were created ranging from 1 to 15. Then these scores were further converted into tertile indicating “poor,” “middle” and “rich” in ascending order. According to Demographic and Health Surveys (DHS), wealth-index is particularly valuable in countries that lack reliable data on income and expenditure (43). However, as explained before, it is derived from a list of assets. If the assets are not reflective of the context, the resultant score may be less than effective in indicating the corresponding socio-economic status (SES). The study also collected information on both income and expenditure, thus, to make the SES stratification representative of the context, a composite SES variable was created by combining income and expenditure with the wealth-index.

Finally, the main independent variables, i.e., changes in the socio-economic characteristics, such as- income, expenditure, working hours, and occupation were indicated as having increased or decreased since being discharged from the hospital and at the time of data collection.

### 2.4. Data analysis

The statistical software “Stata” (version 17) was used to carry out the data analysis. Descriptive analyses were performed and chi-square tests were used to identify group-specific differences where applicable. Multinomial logistic regression analyses were performed to assess the association (unadjusted and adjusted) between the changes in socioeconomic characteristics and mental health-related symptoms. Odds ratios (OR) with a 95% confidence interval (CI) were calculated to assess the strength of the association of mental health-related symptoms and the main independent variables after controlling for the background variables (in the adjusted model). Finally, socio-economic (income, expenditure, and SES) inequalities in experiencing the coexistence of mental health-related symptoms were measured using relative index of inequality (RII), slope index of inequality (SII) and concentration index (CIX). The SII (determined by linear regression) is a weighted measure of inequality that represents the absolute difference in estimated values of a health indicator and RII (computed using a modified Poisson approach) is a weighted measure to quantify the inequality in experiencing any health outcome on a relative scale (44). In this study, for example, values of RII>1 indicates that respondent with higher SES (i.e., rich) are more likely to experience either any one mental health-related symptom, any two symptoms, or all three symptoms, compared to the respondents of lower SES (i.e., poor). Values of RII <1 indicate that the poor are more likely to be exposed to the coexistence of mental health-related symptoms compared to the rich. Again, a positive SII indicates that the coexistence of mental health-related symptoms is likely to increase with the step-by-step increase of respondents' SES level (poor to rich). On the other hand, CIX quantified the degree of inequality of a specific health-related variable over the distribution of another socio-economic related variable of interest, such as respondents' SES, income, or expenditure. If CIX = 0, there is no socio-economic-related inequality. If CIX is negative, the curve lies above the line of equality indicating a disproportionate concentration of the health-related variable among the poor, and vice-versa for the positive value of CIX (44). For all analyses, the significant level was set at P < 0.05. To detect possible collinearity, variance inflation factor (VIF) was used, but no multicollinearity was found among the variables.

## 3. Results

### 3.1. Background characteristics

The demographic and socio-economic characteristics of 481 previously hospitalized patients with symptoms of COVID-19 are presented in Table 1. The mean duration of hospital stay of the participants was 12.2 days. The mean age of the participants, at the time of the survey, was 45.3 *(*±*14.2)* years [men 46.4 years and women 44.1 years]. Six out of 10 participants were men. Most of the participants belonged to nuclear family (53%), resided in their own house (51%), and had more than 12 years of education (48%). Although more than half of the respondents were in paid employment (54%), 41% were from the poor socio-economic group.

**Table 1:** Frequency and percentage distribution of respondents' background characteristics (*N* = 481).

**Background characteristics**	**Frequency (%)**
**Age (in years)**	
*Mean (±SD)*	*45.3 (±14.2)*
**Age groups (in years)**	
≤29	70 (14.5)
30–39	114 (23.7)
40–49	99 (20.6)
50–59	109 (22.7)
60+	89 (18.5)
**Gender**	
Male	290 (60.3)
Female	191 (39.7)
**Household size (members)**	
*Mean (±SD)*	*4.9 (±2.7)*
1–4	254 (52.8)
5–6	145 (30.1)
7 and more	82 (17.1)
**Type of residence**	
Own house	244 (50.7)
Rented house	200 (41.6)
Other	37 (7.7)
**Completed years of education**	
*Mean (±SD)*	*12.3 (±4.8)*
0–5	56 (11.6)
6–10	112 (23.3)
11–12	84 (17.5)
More than 12 years	229 (47.6)

SD, Standard Deviation.

In terms of income and expenditure status of the respondents, median monthly income and expenditure were reported to be 32,000 BDT (minimum: 2,000 BDT, maximum: 700,000 BDT) and 30,000 BDT (minimum: 2,000 BDT, maximum: 600,000 BDT), respectively (Table 2). When asked about the changes in socio-economic status since recovery from COVID-19, 11% of the participants reported changes in occupational status, whereas nearly half reported changes in income (48%) and expenditure (48%). More specifically, of those who reported changes in their income and expenditure, earnings reduced for 92% of the participants (median decrease in income 15,000 BDT; minimum 2,200 BDT, maximum 400,000 BDT), while spending rose for 88% (median increase in expenditure 5,000 BDT; minimum 500 BDT, maximum 20,000 BDT).

**Table 2:** Frequency and percentage distribution of respondents' socio-economic characteristics (*N* = 481 unless specified otherwise).

**Socio-economic characteristics**	**Frequency (%)**
**Occupation**	
In paid employment^a^	261 (54.3)
Not in paid employment^b^	87 (18.1)
Homemaker^c^	133 (27.6)
Monthly income (in BDT^*^) (n = **391)**^d^	
*Median (Minimum, Maximum)*	*32,000 (2,000, 700,000)*
≤20,000	97 (24.8)
20,001–30,000	96 (24.5)
30,001–40,000	46 (11.8)
40,001–60,000	79 (20.2)
60,001+	73 (18.7)
Monthly expenditure (in BDT^*^) (***n*** = **436)**^e^	
*Median (Minimum, Maximum)*	*30,000 (2,000, 600,000)*
≤15,000	90 (20.6)
15,001–25,000	108 (24.8)
25,001–30,000	64 (14.7)
30,001–50,000	110 (25.2)
50,001+	64 (14.7)
**Household socio-economic status (SES) (*****n*** = **382)**	
Poor	156 (40.8)
Middle	112 (29.3)
Rich	114 (29.8)
**Changes in working hours**	
No	359 (74.6)
Yes	122 (25.4)
**Changes in income**	
No	250 (52.0)
Yes^f^	231 (48.0)
*Increased*	*18 (7.8)*
*Decreased*	*213 (92.2)*
**Changes in expenditure**	
No	248 (51.6)
Yes^f^	233 (48.4)
*Increased*	*204 (87.6)*
*Decreased*	*29 (12.4)*
**Changes in occupation**	
No	429 (89.2)
Yes	52 (10.8)

^a^In paid employment includes Govt. employee, private job, business, factory worker, semi-skilled worker/service, unskilled worker, farmer/agriculturist, poultry/livestock, home-based factory, housemaid and technician; ^b^Unemployed includes retired persons, students and unemployed persons; ^c^Homemaker includes unpaid domestic work; ^d^Did not respond: 45 (9.4%), ^e^Did not respond: 90 (18.7%); ^f^Changes in income and expenditure are further classified into increased and decreased. ^*^USD 1 = BDT 84.8 (at the time of survey).

### 3.2. Prevalence of symptoms of depression, anxiety, and stress

**Figure 2:**
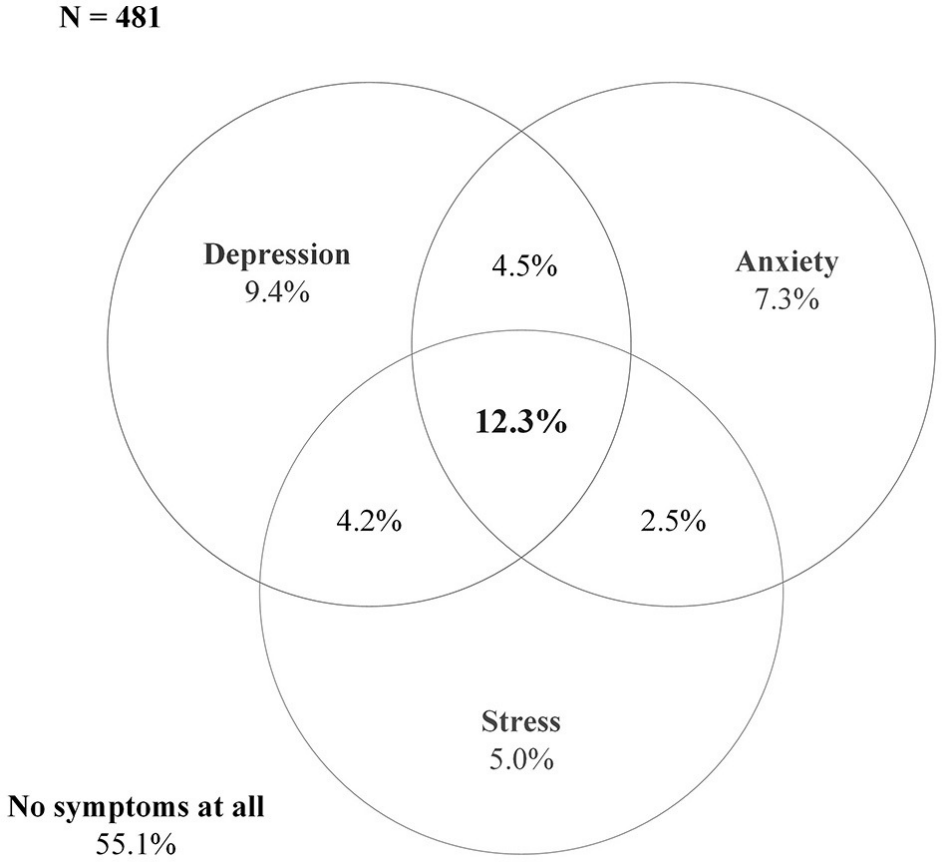
Prevalence and coexistence of symptoms of depression, anxiety, and stress (*N* = 481).

Fifty-five percent of the patients reported no symptoms of depression, anxiety or stress (Figure 2). Among those who reported any of the symptoms, 22% reported any one of the symptoms (i.e., depression 9.4%, anxiety 7.3%, and stress 5%) (Supplementary Figure 1) on average six months since discharge from the hospital. Eleven percent reported having any of the two symptoms simultaneously and 12% reported coexisting symptoms of depression, anxiety, and stress at the same time (Figure 2).

### 3.3. Socio-economic characteristics by the distribution of mental health-related symptoms

The proportional comparison (using bi-variate association) between participants' demographic and socio-economic characteristics, and the distribution of the mental health-related symptoms (depression, anxiety, and stress) is displayed in Supplementary Table 1. The proportion experiencing all three mental health-related symptoms was higher among the age group 30–39 years compared to the older persons aged 60 years and above [32 vs. 15%; *P* (χ^2^) = 0.016]. Significantly more women than men reported the coexistence of all three symptoms [63 vs. 37% *P* (χ^2^) = 0.001]. A significant difference was observed in reporting the coexistence of mental health-related symptoms among participants who reported changes in their working hours [P (χ^2^) = 0.006] and occupation [P (χ^2^) = 0.043] (Supplementary Table 1).

### 3.4. Association between the changes in socio-economic characteristics and mental health-related symptoms

The unadjusted (Supplementary Table 2) and adjusted (Table 3) associations between the changes in socio-economic characteristics and mental health-related symptoms were generated from multinomial logistic regression models. In the adjusted model, considering “no symptom at all” as the reference group, the adjusted odds ratio (AOR) of participants' age (AOR: 0.96, 95% CI: 0.93–0.99; *P* = 0.024) indicates that those with increasing age were less likely to report all three mental health-related symptoms. Participants from the rich SES group (compared to the poor) had lower likelihood of reporting any one mental health-related symptom (AOR: 0.36, 95% CI: 0.16–0.78; *P* = 0.010). In case of gender, women were 2.95 times more likely to report the coexistence of all three symptoms (95% CI: 1.39–5.68; *P* = 0.004) compared to men (Table 3).

**Table 3:** Adjusted multinomial logistic regression to determine the association between the changes in socio-economic characteristics and mental health-related symptoms (Reference group: No symptoms at all) (*n* = 382).

**Background characteristics**	**Depressive, anxiety, and stress symptoms**		
	**Any one symptom AOR [95% CI]**	**Any two symptoms AOR [95% CI]**	**All three symptoms AOR [95% CI]**
**Socio-demographic characteristics**			
***Age** (in years)*	0.99 [0.97–1.01]	0.98 [0.48–2.65]	0.96^*^ [0.93–0.99]
***Gender***			
Men	1 (ref)	1 (ref)	1 (ref)
Women	1.32 [0.72–2.31]	1.27 [0.58–2.74]	2.95^**^ [1.39–5.68]
***Household size** (members)*			
1–4	1 (ref)	1 (ref)	1 (ref)
5–6	0.87 [0.46–1.66]	1.07 [0.45–2.58]	0.80 [0.33–1.96]
7+	0.92 [0.41–2.09]	2.32 [0.85–6.30]	1.08 [0.40–2.94]
***Type of residence***			
Own house	1 (ref)	1 (ref)	1 (ref)
Rented house	1.19 [0.66–2.13]	0.47 [0.21–1.02]	0.54 [0.25–1.18]
Other	0.74 [0.27–2.03]	0.13 [0.02–1.11]	0.12 [0.01–1.07]
***Completed years of education***			
0–5	1 (ref)	1 (ref)	1 (ref)
6–10	1.17 [0.41–3.34]	0.87 [0.23–3.26]	0.47 [0.12–1.76]
11–12	1.02 [0.32–3.23]	1.05 [0.24–4.65]	0.49 [0.12–2.07]
More than 12	1.56 [0.55–4.42]	0.93 [0.25–3.54]	0.82 [0.23–2.87]
**Household SES**			
Poor	1 (ref)	1 (ref)	1 (ref)
Middle	0.65 [0.34–1.22]	0.48 [0.18–1.23]	1.29 [0.49–3.39]
Rich	0.36^*^ [0.16–0.78]	0.43 [0.16–1.16]	1.45 [0.54–3.84]
**Changes in socio-economic status**			
***Changes in occupation***			
No	1 (ref)	1 (ref)	1 (ref)
Yes	0.75 [0.24–2.27]	1.78 [0.48–6.33]	3.04^*^ [1.01–9.08]
***Changes in working hours***			
No change	1 (ref)	1 (ref)	1 (ref)
Yes	2.10^*^ [1.09–3.96]	2.46^*^ [1.07–5.67]	1.25 [0.54–2.88]
***Changes in income***			
No	1 (ref)	1 (ref)	1 (ref)
Yes	1.16 [0.64–2.11]	0.50 [0.22–1.13]	1.36 [0.61–3.03]
***Changes in expenditure***			
No	1 (ref)	1 (ref)	1 (ref)
Yes	1.67 [0.94–2.96]	1.40 [0.64–3.00]	2.46^*^ [1.12–5.37]

ref, Reference category; 95% CI, 95% Confidence Interval; AOR, Adjusted Odds Ratio. Level of significance: ^*^P < 0.05, ^**^P < 0.01.

After adjusting for other covariates (age group, gender, household size, type of residence, years of education, and SES), the model shows that the likelihood of experiencing all three mental health-related symptoms was 3.04 times (95% CI: 1.01–9.08; *P* = 0.048) higher for participants whose occupation had changed since discharge from hospital compared to those with no changes in their occupation. The odds of experiencing any one symptom (AOR: 2.10, 95% CI: 1.09–3.96; *P* = 0.024) and any two symptoms (AOR: 2.46, 95% CI: 1.07–5.67; *P* = 0.035) were significantly higher among participants whose working hours had changed (compared to no changes in working hours). Similarly, participants whose expenditure had changed since discharge from hospital after recovery were more likely to report all three symptoms (AOR: 2.46, 95% CI: 1.12–5.37; *P* = 0.024) compared to participants with no changes.

### 3.5. Income, expenditure, and SES inequality in relation to mental health-related symptoms (*n* = 382)

Table 4 shows the summary measures of inequality in relation to income, expenditure, and SES while experiencing mental health-related symptoms among the study participants. RII values for expenditure and SES in experiencing any one mental health-related symptom were found to be significant (0.84 and 0.74 respectively). It indicates that a move from the lowest to highest expenditure level was associated with a 16% decrease and a move from poor to rich SES level was associated with a 26% decrease in experiencing any one mental health-related symptom. The corresponding SII indicated that one unit change from the lower to higher expenditure level, and poor to rich SES group were significantly associated with 0.04 and 0.06 unit decrease in experiencing any one mental health symptom respectively. Findings from RII and SII are further affirmed through the concentration index (CIX) in relation to expenditure and SES inequalities which suggests that the experience of any one mental health symptom was significantly concentrated among the participants with lower expenditure and poor SES group (Table 4).

**Table 4:** Summary measures of income, expenditure and SES inequality in relation to coexistence of mental health-related symptoms (*n* = 382).

**Participants' socio- economic characteristics**	**Inequality measures**	**Any one symptom**	**Any two symptoms**	**All three symptoms**
Income	RII, RR (95% CI)	0.89 (0.78–1.03)	0.87 (0.72–1.08)	1.05 (0.87–1.22)
	SII, β-coeff. (95% CI)	−0.023 (−0.052–0.004)	−0.012 (−0.032–0.008)	0.005 (−0.016–0.025)
	CIX^*^100 (95% CI)	−7.71 (−16.95–1.52)	−4.61 (−11.61–2.40)	1.63 (−5.61–8.84)
Expenditure	RII, RR (95% CI)	0.84^*^ (0.72–0.95)	0.97 (0.79–1.19)	1.23 (1.00–1.52)
	SII, β-coeff. (95% CI)	−0.040^**^(−0.068–0.011)	−0.003 (−0.025–0.018)	0.022 (−0.001–0.044)
	CIX^*^100 (95% CI)	−11.98^*^ (−21.18–2.80)	−0.92 (−7.94–6.02)	7.31 (1.01–16.53)
SES	RII, RR (95% CI)	0.74^*^ (0.58–0.95)	0.85 (0.60–1.22)	1.25 (0.90–1.73)
	SII, β-coeff. (95% CI)	−0.062^*^ (−0.109–0.014)	−0.016 (−0.052–0.019)	0.025 (−0.013–0.063)
	CIX^*^100 (95% CI)	−10.88^*^ (−19.72–12.05)	−3.16 (−9.89–3.57)	4.58 (−2.36–11.52)

RII, Relative Inequality index; SII, Slope index of inequality; CIX, Concentration index; RR, Risk Ratio; 95% CI, 95% Confidence Interval.

Level of significance: ^*^P < 0.05, ^**^P < 0.01.

## 4. Discussion

This is the first study in Bangladesh to investigate the prevalence of mental health-related symptoms of previously hospitalized patients with symptoms of COVID-19 and its association with socio-economic changes due to the pandemic. Using a validated scale (DASS-21) (42), the findings indicate about 45% of the study participants reported symptoms of depression, anxiety and/or stress (depression 9.4%, anxiety 7.3%, and stress 5%), and about 23% coexistence of any two or all three symptoms. A recently published systematic review of studies from LMICs reported the burden of mental health-related symptoms among previously hospitalized COVID-19 patients (14). According to the review, within a period of 3–6 months after being discharged from the hospital, 5–23% reported depressive symptoms, 7–21% anxiety symptoms, 6% stress, and 5% a new mood disorder (14). This is similar to the findings of our study. Patients with COVID-19 who were hospitalized for nearly 2 weeks might experience significant mental health-related symptoms after discharge from the hospital due to problems related to lock-down (such as- mobility restrictions in the streets and marketplaces, absence of social activities, and increased burden of household chores for women) (14, 45). It is important to note that, the pre-COVID-19 burden of mental health-related morbidity in Bangladesh has been reported to be 17% (depression 6.7% and anxiety disorder 4.5%) in the National Mental Health Survey (2019) of Bangladesh (46). Research indicated that a considerable rise in the burden of mental health-related morbidity in Bangladesh may be related to COVID-19 (20, 22, 31).

The findings of this study indicate that participants with the coexistence of all three symptoms were more likely to be of the age group 30–39 years, women, reported changes in occupation and in monthly expenditure. Other studies from Bangladesh (10, 31), China (47), Japan (48), and Turkey (23) also reported that during the COVID-19 pandemic, women reported significantly higher psychological distress compared to men. There is evidence that in a post-disaster context, women are more vulnerable to mental health-related morbidities due to greater workload in the household, increased responsibilities toward family members, and high incidence of violence (49). According to the findings of our study, older persons were less likely to report all three mental health-related symptoms than their younger counterparts. Studies from Japan (48) and Pakistan (24) also reported that the level of mental health-related symptoms appeared to decline with increasing age. Based on the recent evidence, possible explanation to this can be that young adults are more exposed to social media than the older ones, and tend to obtain a large amount of COVID-19 related information (such as- infected and death rate per days) from internet and social media, which consequently may lead to fear, panic, and other psychological problems among them (24, 47). Additionally, the younger adults may also have been anxious about delayed entry to the job market as a result of the pandemic (50).

It is important to note that this study showed that almost half of the study participants reported changes in their socio-economic situations. A quarter of the study participants reported changes in their working hours and almost every tenth participant had changed occupation. One of the studies from Bangladesh found that at least one family member in three out of 10 households lost source of income due to the disruptions caused by COVID-19 and 41% of respondents started searching for a new job because of changes in their work schedule (51). During the pandemic, others have reported a substantial decline in income and savings (38, 39, 52), in some cases as much as 70 and 60% respectively (53). There is evidence that the COVID-19 pandemic led to restricted economic activities which in turn forced many to exhaust their savings and/or sell assets resulting in a nationwide economic crisis (53–55). According to the findings of this study, participants who reported experiencing one mental health-related symptom were more likely to be from the lower socio-economic group. Recent evidence indicated higher socio-economic status to be associated with less likelihood of developing mental health-related morbidities (19). Other studies from LMIC contexts have presented similar findings (14, 56–58). In a non-COVID scenario, often people from the lowest SES groups report significantly higher rates of any mental health-related problems than those in higher SES groups (59). A study from Bangladesh reported that 63% of poor and vulnerable households reduced food consumption, 50% sought financial help from friends and 22% of the households searched for more work due to lowered capacity to meet household expenditure resulting from income loss (51). Thus, based on the findings of this study and existing evidence, it is critical to further investigate the nature of the association between economic struggle and post-COVID mental health-related consequences in Bangladesh (and in LMICs) for appropriate response in the future.

One of the limitations of the study is the high non-response rate (49%). Since it was a telephone-based survey, some respondents could not be reached over phone, some did not agree to participate, and few were available at times (e.g., late nights) which was impossible for the data collectors to conduct interviews. Secondly, a few respondents were reluctant to provide their socio-economic information, especially regarding their monthly income. Thus, a considerable proportion of socio-economic data (20%) was missing. Thirdly, no information on vaccination and personal history of pre-existing psychiatric disorders of the study participants were available in this study. At the time of data collection, COVID-19 vaccination program in Bangladesh had only started. In addition, availability and utilization of mental health care in Bangladesh is still inadequate and it is not possible to compare the burden of mental health issues reported in this study with personal history of pre-existing psychiatric disorders. Fourthly, the mental health related symptoms in this study were assessed by a rater-administered scale over the phone. Due to the restrictions in mobility and physical distancing the interviews were conducted over the phone, and actual psychiatric screening and the use of diagnostic scales or collection of psychiatric anamnestic data was not possible. Instead, we used a validated scale to assess the symptoms of depression, anxiety, and stress. However, it is important to note that DASS-21 is a widely acknowledged and validated tool for documenting mental health-related symptoms, and the data collectors were rigorously trained in administering survey instruments *via* telephone. Given the challenges of capturing mental health-related information and regulation of lockdown in terms of mobility and physical distancing, DASS-21 and data collection over phone have been tested and acknowledged to be a valuable approach (60, 61). Finally, this study included only the previously hospitalized patients with COVID-19. Hence, the findings of this study cannot be generalized to the general population.

## 5. Conclusion

The study identifies specific groups who are particularly vulnerable in terms of mental health-related symptoms among those previously hospitalized with COVID-19-like symptoms. It is expected that this study will help in addressing mental health-related challenges after recovery from COVID-19 among the identified vulnerable groups through relevant community-based and clinical response including counseling services in Bangladesh and similar LMIC contexts and contribute to further implementation research in this regard. However, clinical research is needed to determine mental health-related diagnoses among the general population in the context of post-COVID. It is also important to gain insight about the mental health status of the rural population in the country in relation to the pandemic.

## Data availability statement

The raw data supporting the conclusions of this article will be made available by the authors, without undue reservation.

## Ethics statement

The studies involving human participants were reviewed and approved by Bangladesh Medical Research Council (Reference no. 35115102020). The Ethics Committee waived the requirement of written informed consent for participation.

## Author contributions

AA, TA, and ZK conceptualized the study. AP supervised data collection. AA analyzed the data and drafted the manuscript. AA, TA, AN, AP, SD, and ZK interpreted the data. All authors read and reviewed the draft and approved the final manuscript for publication.

## Supplementary material

Supplementary Figure 1 Coexistence of symptoms of depression, anxiety, and stress (*N* = 481).

Supplementary Table 1 Proportional comparison of respondents' background characteristics and number of mental health-related symptoms (*N* = 481).

Supplementary Table 2 Unadjusted multinomial logistic regression to determine the association between the changes in socio-economic characteristics and mental health-related symptoms (Reference group: No symptoms at all) (*n* = 382).
