# Supplementary figures and images for: Evidence of mental health-related morbidities and its association with socio-economic status among previously hospitalized patients with symptoms of COVID-19 in Bangladesh
Source: Front Public Health. 2023 Feb 24;11:1132136. doi: 10.3389/fpubh.2023.1132136 (PMC9998677; doi:10.3389/fpubh.2023.1132136)

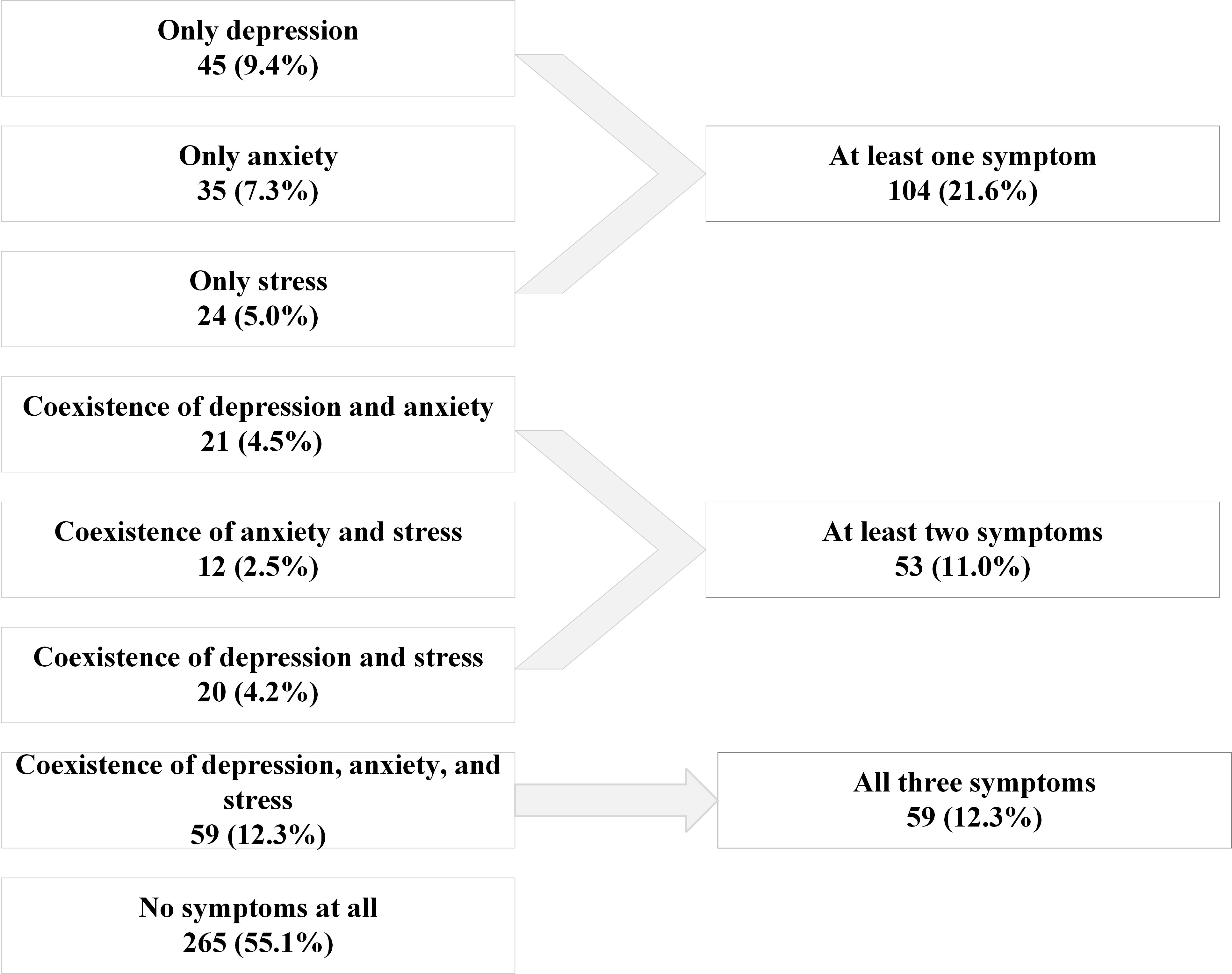

Supplement: Supplementary Figure 1 — Coexistence of symptoms of depression, anxiety, and stress (N = 481). [file Image_1.TIF]
