# Supplementary material for: Evidence of mental health-related morbidities and its association with socio-economic status among previously hospitalized patients with symptoms of COVID-19 in Bangladesh
Source: Front Public Health. 2023 Feb 24;11:1132136. doi: 10.3389/fpubh.2023.1132136 (PMC9998677; doi:10.3389/fpubh.2023.1132136)
Supplement: Supplementary Table 1 — Proportional comparison of respondents' background characteristics and number of mental health-related symptoms (N = 481). [file Table_1.DOCX]

**Supplementary Materials** (tables)

**Supplementary Table 1: Proportional comparison of respondents’ background characteristics and number of mental health-related symptoms** (N = 481)

| Variables | Symptoms of depression, anxiety, and stress  n (%) | | | |  |
| --- | --- | --- | --- | --- | --- |
|  | No symptoms | Any one symptom | Any two symptoms | All 3 symptoms | *P (ꭓ^2^) - values* |
| **Socio-demographic characteristics** | | |  |  |  |
| ***Age groups*** |  |  |  |  | *0.016* |
| <= 29 | 35 (13.2) | 18 (17.3) | 5 (9.4) | 12 (20.3) |  |
| 30 – 39 | 54 (20.4) | 26 (25.0) | 15 (28.3) | 19 (32.2) |  |
| 40 – 49 | 48 (18.1) | 27 (26.0) | 14 (26.4) | 10 (16.9) |  |
| 50 – 59 | 65 (24.5) | 19 (18.3) | 16 (30.2) | 9 (15.2) |  |
| 60+ | 63 (23.8) | 14 (13.5) | 3 (5.7) | 9 (15.2) |  |
| ***Gender*** |  |  |  |  | *0.001* |
| Male | 175 (66.0) | 64 (61.5) | 29 (54.7) | 22 (37.3) |  |
| Female | 90 (34.0) | 40 (38.5) | 24 (45.3) | 37 (62.7) |  |
| ***Household size*** | |  |  |  | *0.958* |
| 1 – 4 | 141 (53.2) | 55 (52.9) | 26 (49.1) | 32 (54.2) |  |
| 5 – 6 | 80 (30.2) | 33 (31.7) | 15 (28.3) | 17 (28.8) |  |
| 7 and over | 44 (16.6) | 16 (15.4) | 12 (22.6) | 10 (17.0) |  |
| ***Residence type*** | |  |  |  | *0.111* |
| Own house | 136 (51.3) | 43 (41.3) | 32 (60.4) | 33 (55.9) |  |
| Rented house | 107 (40.4) | 49 (47.1) | 19 (35.9) | 25 (42.4) |  |
| Others | 22 (8.3) | 12 (11.5) | 2 (3.8) | 1 (1.7) |  |
| ***Completed years of education*** | |  |  |  | *0.922* |
| 0 – 5 | 33 (58.9) | 10 (17.9) | 6 (10.7) | 7 (12.5) |  |
| 6 – 10 | 61 (54.5) | 23 (20.5) | 15 (13.4) | 13 (11.6) |  |
| 11 – 12 | 48 (57.1) | 15 (17.9) | 11 (13.1) | 10 (11.9) |  |
| More than 12 | 123 (53.7) | 56 (24.5) | 21 (9.2) | 20 (12.7) |  |
| **Socio-economic characteristics** | | |  |  |  |
| ***Household SES*** (n = 382) | |  |  |  | *0.185* |
| Poor | 80 (37.0) | 41 (50.6) | 20 (48.8) | 15 (34.1) |  |
| Middle | 66 (30.6) | 24 (29.6) | 10 (24.4) | 12 (27.3) |  |
| Rich | 70 (32.4) | 16 (19.7) | 11 (26.8) | 17 (38.6) |  |
| ***Occupation*** |  |  |  |  | *0.027* |
| Currently paid employment | 158 (59.6) | 53 (51.0) | 30 (56.6) | 20 (33.9) |  |
| Not paid employment | 45 (17.0) | 20 (19.2) | 8 (15.1) | 14 (23.7) |  |
| Homemaker | 62 (23.4) | 31 (29.8) | 15 (28.3) | 25 (42.4) |  |
| ***Monthly expenditure*** (n = 382) (in BDT*) | | |  |  | *0.201* |
| <= 15,000 | 41 (19.0) | 19 (23.46) | 9 (22.0) | 5 (11.4) |  |
| 15,001 – 25,000 | 45 (20.8) | 27 (33.3) | 10 (24.4) | 10 (22.7) |  |
| 25,001 – 30,000 | 33 (15.3) | 10 (12.4) | 5 (12.2) | 7 (15.9) |  |
| 30,001 – 50,000 | 63 (29.2) | 17 (21.0) | 10 (24.4) | 9 (20.4) |  |
| 50,001 + | 34 (15.7) | 8 (9.9) | 7 (17.1) | 13 (29.5) |  |
| ***Monthly income*** (n = 382) (in BDT*) | | |  |  | *0.575* |
| <= 20,000 | 45 (20.8) | 24 (29.6) | 14 (34.2) | 12 (27.3) |  |
| 20,001 – 30,000 | 52 (24.1) | 22 (27.2) | 10 (24.4) | 9 (20.4) |  |
| 30,001 – 40,000 | 28 (13.0) | 9 (11.1) | 4 (9.8) | 5 (11.4) |  |
| 40,001 – 60,000 | 51 (23.6) | 15 (18.5) | 6 (14.6) | 6 (13.6) |  |
| 60,001+ | 40 (18.5) | 11 (13.6) | 7 (17.1) | 12 (27.3) |  |
| ***Changes in working hours*** | |  |  |  | *0.006* |
| No change | 213 (80.4) | 72 (69.2) | 32 (60.4) | 42 (71.2) |  |
| Yes | 52 (19.6) | 32 (30.8) | 21 (39.6) | 17 (28.8) |  |
| ***Changes in Income*** | |  |  |  | *0.06* |
| No | 149 (56.2) | 51 (49.0) | 28 (52.8) | 22 (37.3) |  |
| Yes | 116 (43.8) | 53 (51.0) | 25 (47.2) | 37 (62.7) |  |
| ***Changes in expenditure*** | |  |  |  | *0.06* |
| No | 150 (56.6) | 49 (47.1) | 26 (49.1) | 23 (39.0) |  |
| Yes | 115 (43.4) | 55 (52.9) | 27 (50.9) | 36 (61.0) |  |
| ***Changes in occupation*** | |  |  |  | *0.043* |
| No | 244 (92.1) | 93 (89.4) | 44 (83.0) | 48 (81.4) |  |
| Yes | 21 (7.9) | 11 (10.6) | 9 (17.0) | 11 (18.6) |  |
| **Total (n)** | **265** | **104** | **53** | **59** |  |

*USD 1 = BDT 84.8 (at the time of survey);

*P (ꭓ^2^) – values,* obtained from chi-2 estimation

**Supplementary Table 2: Unadjusted multinomial logistic regression to determine the association between the changes in socio-economic characteristics and mental health-related symptoms (Reference group: No symptoms at all)** (n = 382)

| **Variables** | **Symptoms of depression, anxiety and stress** | | |
| --- | --- | --- | --- |
|  | **Any one symptom**  **UOR, 95% CI** | **Any two symptoms**  **UOR, 95% CI** | **All three symptoms**  **UOR, 95% CI** |
| **Changes in occupation** |  |  |  |
| No | 1 (ref) | 1 (ref) | 1 (ref) |
| Yes | 0.77 [0.26 – 2.27] | 1.67 [0.50 – 5.58] | 3.01* [1.10 – 8.31] |
| **Changes in working hours** |  |  |  |
| No | 1 (ref) | 1 (ref) | 1 (ref) |
| Yes | 2.02* [1.09 – 3.78] | 2.56* [1.17 – 5.59] | 1.33 [0.60 – 2.92] |
| **Changes in Income** |  |  |  |
| No | 1 (ref) | 1 (ref) | 1 (ref) |
| Yes | 1.04 [0.59 – 1.85] | 0.55 [0.25 – 1.20] | 1.22 [0.57 – 2.60] |
| **Changes in expenditure** |  |  |  |
| No | 1 (ref) | 1 (ref) | 1 (ref) |
| Yes | 1.81* [1.03 – 3.16] | 1.49 [0.72 – 3.09] | 2.07* [1.02 – 3.30] |

Ref: Reference category; 95% CI: 95% Confidence Interval; UOR: Un-adjusted Odds Ratio

Level of significance: *P<0.05, **P<0.01, ***P<0.001
